# Screening of anticancer drugs to detect drug‐induced interstitial pneumonia using the accumulated data in the electronic medical record

**DOI:** 10.1002/prp2.421

**Published:** 2018-07-12

**Authors:** Yoshie Shimai, Toshihiro Takeda, Katsuki Okada, Shirou Manabe, Kei Teramoto, Naoki Mihara, Yasushi Matsumura

**Affiliations:** ^1^ Department of Medical Informatics Osaka University Graduate School of Medicine Suita Japan; ^2^ Division of Medical Informatics Tottori University Hospital Suita Japan; ^3^ Department of Medical Informatics National Cancer Center Hospital Suita Japan

**Keywords:** adverse drug reaction, electronic medical record, imaging report, interstitial pneumonia, natural language processing

## Abstract

Because drug‐induced interstitial pneumonia (DIP) is a serious adverse drug reaction, its quantitative risk with individual medications should be taken into due consideration when selecting a medicine. We developed an algorithm to detect DIP using medical record data accumulated in a hospital. Chest computed tomography (CT) is mainly used for the diagnosis of IP, and chest X‐ray reports, KL‐6, and SP‐D values are used to support the diagnosis. The presence of IP in the reports was assessed by a method using natural language‐processing, in which IP was estimated according to the product of the likelihood ratio of characteristic keywords in each report. The sensitivity and the specificity of the method for chest CT reports were 0.92 and 0.97, while those for chest X‐ray reports were 0.83 and 1, respectively. The occurrence of DIP was estimated by the patterns of presence of IP before, during, and after the administration of the target medicine. The occurrence rate of DIP in cases administered Gefitinib; Methotrexate (MTX); Tegafur, Gimeracil, and Oteracil potassium (TS‐1); and Tegafur and Uracil (UTF) was 6.0%, 2.3%, 1.4%, and 0.7%, respectively. The estimated DIP cases were checked by having the medical records independently reviewed by medical doctors. By chart review, the positive predictive values of DIP against Gefitinib, MTX, TS‐1, and UFT were 69.2%, 44.4%, 58.6%, and 77.8%, respectively. Although the cases extracted by this method included some that did not have DIP, this method can estimate the relative risk of DIP between medicines.

**Abbreviations**
DIP: drug‐induced interstitial pneumonia
IP: interstitial pneumonia
MTX: Methotrexate
TS‐1: Tegafur, Gimeracil, and Oteracil potassium
UFT: Tegafur and Uracil

## 1 INTRODUCTION

The quantitative risk for adverse reaction associated with individual medications should be strongly considered when selecting a medicine. The risks of adverse reactions for medicines are evaluated in clinical trials before the drugs are introduced into the market.^1^, ^2^, ^3^ However, because the number of subjects in clinical trials is limited, information regarding adverse reactions generated in clinical trials may be inadequate.^4^, ^5^, ^6^ Therefore, postmarket pharmacovigilance is required to ensure drug safety. At present, spontaneous reporting is the major method for gathering information about adverse events. This method is effective for detecting signals of adverse reactions of medicines; however, it is impossible to estimate the rate of occurrence of each adverse reaction because the denominator cannot be acquired.^7^, ^8^, ^9^, ^10^, ^11^, ^12^

Recently, many hospitals have introduced electronic medical record (EMR) systems. Some of these systems include a clinical data warehouse (CDW) for the secondary use of the clinical data, which includes data relating to drug safety.^13^, ^14^, ^15^, ^16^, ^17^, ^18^, ^19^, ^20^ Several researchers have attempted to detect adverse drug events using long‐term inpatients’ laboratory or pharmacy data in EMR.^21^ For example, Cheetham et al. developed an automated causality assessment algorithm to identify drug‐induced liver injury in EMR data using the Roussel Uclaf Causality Assessment Method (RUCAM).^22^ In our previous study, adverse events were detected based on the changes in the blood test results, and the adverse reactions of a designated drug were estimated by the chronological relationship between the occurrence of the events and the drug administration.^23^ However, the occurrence of adverse events that can be assessed by numeric data, such as blood test results, is relatively infrequent. Many of them must be assessed by analyzing free‐text data in EMR.

Interstitial pneumonia (IP) is one of the most serious drug‐induced adverse reactions and can potentially lead to the death of the patient.^24^ Chest computed tomography (CT) is mainly used for the diagnosis of IP. Chest X‐ray can diagnose IP in severe cases but not in the early stage. The sialylated carbohydrate antigen KL‐6 (KL‐6) and surfactant protein D (SP‐D) levels are elevated in some IP cases. Thus, chest X‐ray findings and the results of KL‐6 and SP‐D are helpful for the diagnosis of IP. Because radiologists usually write their image reports in free‐text form, natural language‐processing (NLP) must be used for their analysis.^25^, ^26^, ^27^, ^28^, ^29^ In a previous study, McCowan et al. extracted cancer staging information from pathology reports using support vector machines (SVMs).^30^ Dublin et al. identified pneumonia from radiology reports using logistic regression,^31^ while Pham et al. detected thromboembolic diseases or pulmonary embolism from radiology reports using Naive Bayes.^32^

In this study, we developed an algorithm to estimate the occurrence rate of drug‐induced IP (DIP) of a designated medicine by assessing the certainty of IP from imaging reports and blood test results in EMR before, during, and after the administration of the medicine.

## 2 MATERIALS AND METHODS

This study protocol was approved by the Ethics Review Board of Osaka University Medical Hospital (Approval No. 13531, May 8th, 2014).

We initially developed a method for detecting the occurrence of IP from the text data of chest CT and chest X‐ray reports and the data of the KL‐6 and SP‐D. Next, we devised a method to estimate DIP induced by a designated medicine.

### 2.1 Method for detecting IP from reports of chest CT and chest X‐ray

**Figure 1:**
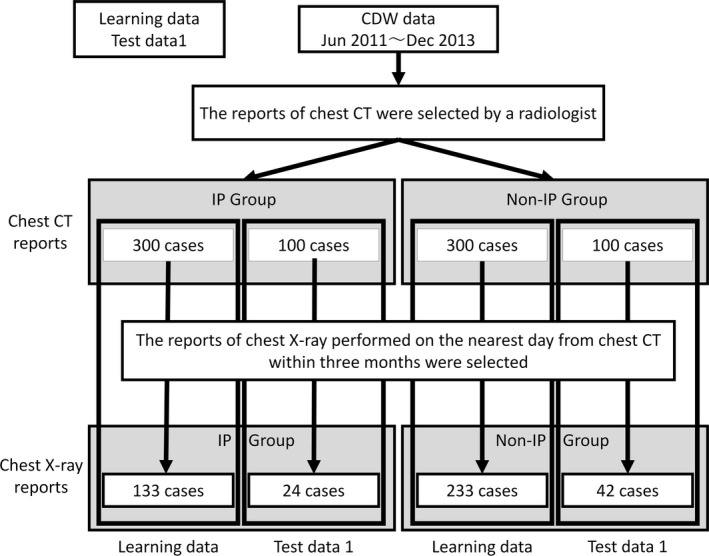
Dataset for learning data and test data 1. The same numbers of CT reports for the IP and Non‐IP groups were selected by a radiologist from clinical data warehouse data. The CT reports in each group were allocated to the learning dataset and test data 1. Reports for X‐ray performed on the day nearest to the CT examination within 3 months were selected in the same patients in the learning data and test data 1

**Figure 2:**
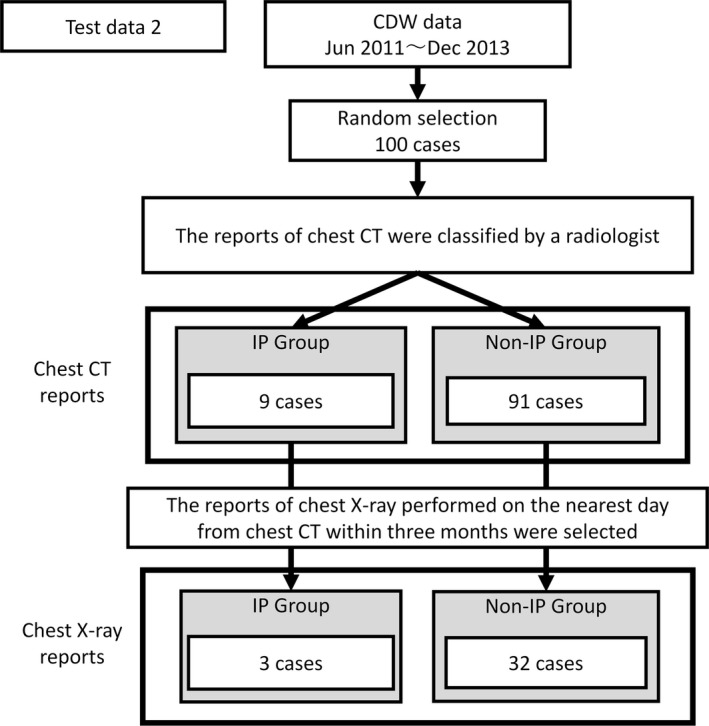
Dataset for test data 2. One hundred CT reports were selected at random for test data 2. Test data 2 was classified into IP and Non‐IP Group by a radiologist. X‐ray reports which was performed on the nearest day from CT examination within 3 months were selected for test data 2 of X‐ray

We used the data contained in the CDW of Osaka University Medial Hospital from January 1, 2010 to December 31, 2013. We selected 400 chest CT reports in patients with IP and 400 chest CT reports in patients without IP. The diagnoses were made by a radiologist. Among these cases, 300 reports in each group were allocated to the learning data, and 100 were allocated to the testing data (test data 1). In addition, we selected 100 chest CT reports at random (test data 2) that were not being used for the learning data or test data 1. We also selected the reports of chest X‐ray performed nearest the examination date for chest CT within 3 months in the same patients in the learning data, test data 1, and test data 2, respectively. The allocated numbers for the learning data and test data 1 are shown in Figure 1, with those for test data 2 in Figure 2.

The chest CT and chest X‐ray reports are written in Japanese and consist of finding and diagnosis fields, which were independently assessed. If IP was judged in either field, IP was regarded as having been diagnosed by the reports.

In the diagnosis field, we searched for the keyword “interstitial pneumonia.” If IP was definitively diagnosed, a flag for IP was set on the report. A synonym or detailed diagnosis of IP, such as “usual interstitial pneumonia (UIP)” or “acute interstitial pneumonia (AIP)” was regarded as IP. There were some diseases with findings similar to those of IP, such as “edema of the lungs” and “viral pneumonia.” There were also cases of IP but not DIP, such as “lymphocytic interstitial pneumonia (LIP)” and “respiratory bronchiolitis‐associated interstitial lung disease (RB‐ILD).” In these cases, DIP was denied and the flag for IP removed.

In the finding field, we assessed the certainty of IP based on Bayes’ theorem. Posterior odds were calculated by the prior odds and likelihood ratio obtained by the product of the likelihood ratios of the keywords written in a report on the assumption of independence of the appearance of the key words.

Posterior odds=prior odds×likelihood ratio

Because prior odds were a constant, we defined the IP score as the product of the likelihood ratios of the keywords, representing the certainty of the reports diagnosing IP.

We extracted keywords from the text data of the chest CT reports in the learning dataset. For the morphological analysis, we used the KH Coder to collect keywords.^33^ Abbreviations and detailed words, such as “UIP” and “AIP”, and synonymous words, such as “frosted glass” and “ground glass” were regarded as the same keywords. The words that appeared in more than 10 reports of IP were selected, and the frequency with which each keyword appeared in the reports of patients with and without IP in the learning dataset was determined. The positive and negative likelihood ratios were then calculated. We adopted the same method for the chest X‐ray learning dataset to obtain the keywords and their likelihood ratios.

When a negative word, such as “not accepted” or “not confirmed” appeared within 15 letters of a keyword, the keyword was regarded as absent. To exclude keywords modifying organs other than lung (eg, “liver cyst” or “aortic calcification”), we checked whether or not a different organ name was included within ± 15 letters of the keyword in a sentence.

To evaluate the IP score for detecting reports with IP, we plotted a receiver operating characteristic (ROC) curve of the score by changing the cut‐off point and obtained the nearest cut‐off value to point (0, 1) on the ROC curve using test data 1. We also evaluated the IP score using test data 2.

### 2.2 Detection of DIP caused by anticancer drugs

In this study, we evaluated the risk of DIP with Gefitinib; Methotrexate (MTX); Tegafur, Gimeracil, and Oteracil potassium (TS‐1); and Tegafur and Uracil (UTF) from the reports of chest CT and chest X‐ray and the level of KL‐6 and SP‐D. The subjects were patients treated with these anticancer drugs in the period from January 1, 2000 to December 31, 2014. The reports of chest CT and chest X‐ray were judged to be IP‐positive or IP‐negative by the above‐mentioned method. The KL‐6 and SP‐D levels were judged to be positive or negative according to the upper limit of normal values of each test.

If the nonadministration period of the designated medicine in the order data was within 30 days, the order records were combined, and the start date of the initial order data was set as the start date of the medicine, while the end date of the last order data was set as the end date. The preadministration period was defined as the day before the start date of the designated medicine. The administration period was defined as the day from the start date to 30 days after the end date of the medicine. The postadministration period was defined as starting the day after the administration period.

The presence of IP was assessed by the CT reports drafted in each period. If CT was not performed, X‐ray findings and the level of KL‐6 or SP‐D were used. If the diagnosis of these examination matched that determined by chest CT in the same period, and if the diagnosis with the same examination was different in other periods, we adopted this diagnosis (Figure S1). The occurrence of DIP was estimated by the “positive,” “negative,” and “not available” patterns of the preadministration, administration, and postadministration periods (Table 1). The confident degree was categorized in “definitive,” “strongly suspected,” “weakly suspected,” “negative,” and “not determined.” Cases that were IP‐positive in administration period and IP‐negative in pre‐ and postadministration period were judged to be “definitive” DIP. Cases that were IP‐negative in preadministration period and IP‐positive in administration period with no available pattern in postadministration period, or cases that IP‐positive in administration period and IP‐negative in postadministration period with no available pattern in preadministration period were estimated as “strongly suspected” DIP. Cases that were IP‐positive in administration period but with no available pattern in pre‐ and postadministration periods were judged to be “weakly suspected” DIP.

**Table 1:** Detection of DIP based on the pattern of the presence of IP before, during, and after drug administration

Drug administration			Judgment	Drug administration			Judgment
Before	During	After		Before	During	After	
−	−	−	Negative	+	NA	−	Negative
−	−	+	Negative	+	NA	+	Negative
−	+	−	Definitive	−	−	NA	Negative
−	+	+	Strongly suspected	−	+	NA	Strongly suspected
+	−	−	Negative	+	−	NA	Negative
+	−	+	Negative	+	+	NA	Negative
+	+	−	Negative	NA	NA	+	ND
+	+	+	Negative	NA	NA	−	ND
NA	−	−	Negative	NA	+	NA	Weakly suspected
NA	−	+	Negative	NA	−	NA	Negative
NA	+	−	Strongly suspected	+	NA	NA	Negative
NA	+	+	ND	−	NA	NA	ND
−	NA	−	ND	NA	NA	NA	ND
−	NA	+	ND				

NA, Not available; ND, Not determined.

To evaluate the results of the algorithm, 3 doctors checked the medical records of the patients estimated as suffering from more than “weakly suspected” DIP. Each doctor independently judged the cases in the category of DIP, not DIP, or not determined. If 2 or more doctors made a diagnosis in the same category, this category was adopted as the overall judgment. If the judgments of the 3 doctors were all completely different, the overall judgment was set as “not determined.”

## 3 RESULTS

### 3.1 Analyses of chest CT and chest X‐ray findings

The list of the keywords selected from the chest CT reports with positive and negative likelihood ratios calculated from the data in the learning dataset are shown in Table S1. Those from the chest X‐ray reports are shown in Table S2.

The sensitivity and specificity of this method applied to CT reports were 0.92 and 0.97, respectively, using the cut‐off value 0.06 (Table 2). When applied to chest X‐ray reports, the sensitivity was 0.83 and the specificity was 1 using the cut‐off value 0.012. When this method was applied to the test data 2, the sensitivity and specificity were 0.89 and 0.99 for chest CT, and 0.67 and 0.97 for chest X‐ray.

**Table 2:** The accuracy of identifying the presence of IP

		Diagnosis by the radiologist		
		IP	Non‐IP	Total
Test data 1, chest CT reports				
Machine analysis	IP	92	3	95
	Non‐IP	8	97	105
	Total	100	100	200
Test data 1, chest X‐ray reports				
Machine analysis	IP	25	0	25
	Non‐IP	5	43	48
	Total	30	43	73
Test data 2, chest CT reports				
Machine analysis	IP	8	1	9
	Non‐IP	1	90	91
	Total	9	91	100
Test data 2, chest X‐ray reports				
Machine analysis	IP	2	1	3
	Non‐IP	1	31	32
	Total	3	32	35

IP, interstitial pneumonia.

### 3.2 Detection of DIP caused by anticancer drugs

The numbers of patients who received Gefitinib, MTX, TS‐1, and UFT were 217, 390, 2088, and 1333, respectively (Table 3). When “definitive,” “strongly suspected,” and “weakly suspected” statuses were deemed to be DIP, the occurrence rate of DIP for Gefitinib, MTX, TS‐1, and UFT was 6.0%, 2.3%, 1.4%, and 0.7%, respectively.

**Table 3:** The number of patients with DIP caused by anticancer drugs

Detection of DIP	Gefitinib	MTX	TS‐1	UFT
Definitive	3	0	13	2
Strongly suspected	10	2	10	4
Weakly suspected	0	7	6	3
Negatively suspected	27	170	873	170
Negative	149	84	846	430
Not determined	28	127	340	724
Total	217	390	2,088	1,333

DIP, drug‐induced interstitial pneumonia; MTX, Methotrexate; TS‐1, Tegafur, Gimeracil, and Oteracil potassium; UFT, Tegafur and Uracil.

### 3.3 The evaluation of DIP according to the medical records

The results of the evaluation of the algorithm by chart review are shown in Table 4. Of the 13 patients who took Gefitinib and were assessed as having DIP, 9 were determined to have DIP and 4 were not, giving a positive predictive value of 69.2%. The positive predictive values with MTX, TS‐1, and UFT were 44.4%, 58.6%, and 77.8%, respectively. The occurrence rate of DIP of Gefitinib, MTX, TS‐1, and UFT calculated from the data ultimately determined by doctors were 4.1%, 1.0%, 0.8%, and 0.6%, respectively.

**Table 4:** Number of patients determined as having DIP by a chart review

Machine analysis		Result by chart review		
Judgment	Total number of the patients	DIP	Non‐DIP	Not determined
Gefitinib (Diagnostic accuracy: 69.2%)				
Definitive	3	1	2	0
Strongly suspected	10	8	2	0
Weakly suspected	0	0	0	0
Total	13	9	4	0
MTX (Diagnostic accuracy: 44.4%)				
Definitive	0	0	0	0
Strongly suspected	2	0	2	0
Weakly suspected	7	4	3	0
Total	9	4	5	0
TS‐1 (Diagnostic accuracy: 58.6%)				
Definitive	13	8	5	0
Strongly suspected	10	7	3	0
Weakly suspected	6	2	4	0
Total	29	17	12	0
UFT (Diagnostic accuracy: 77.8%)				
Definitive	2	1	0	1
Strongly suspected	4	3	1	0
Weakly suspected	3	3	0	0
Total	9	7	1	1

DIP, drug‐induced interstitial pneumonia; MTX, Methotrexate; TS‐1, Tegafur, Gimeracil, and Oteracil potassium; UFT, Tegafur and Uracil.

To determine the reasons for the incorrect estimation of the algorithm, we closely checked the medical records of the 22 patients in whom the results of the algorithm were judged to be incorrect (Table 5). The diagnosis on the CT report was incorrect in 1 patient each for Gefitinib, MTX, and TS‐1. Among the other 19 cases, 13 were more likely to be suffering from other diseases, such as carcinomatous lymphangiosis, emphysema, or a worsening of the primary disease, and 6 were deniable because the chronological relationship of occurrence of IP and drug administration were incompatible with DIP.

**Table 5:** The causes of misjudgment

The causes of misjudgments	Gefitinib	MTX	TS‐1	UFT
The determination of IP was incorrect.	1	1	1	0
The case was finally diagnosed with other disease^a^	1	4	8	0
Chronological relation was Incompatible with DIP	2	0	3	1
Total	4	5	12	1

IP, interstitial pneumonia; DIP, drug‐induced interstitial pneumonia; MTX, Methotrexate; TS‐1, Tegafur, Gimeracil, and Oteracil potassium; UFT, Tegafur and Uracil.

a The final diagnosis: carcinomatous lymphangiosis, emphysema, worsening of primary disease etc.

## 4 DISCUSSION

In this study, we tried to detect DIP from the data in medical records. Because chest CT is a key examination for the diagnosis of IP, reports of chest CT—which are written in free text—had to be analyzed. We therefore developed a method of detecting such reports in which IP was diagnosed.

Theoretically, diagnosis data should be sufficient to detect IP. In actual image reports, however, descriptions about IP are sometimes written only in the findings field. Therefore, we analyzed both the diagnosis and findings of image reports. For the field analysis, we assessed the presence or absence of characteristic keywords for IP and obtained an IP score. However, to detect the presence of a keyword in a report, it is not enough to simply check for the existence of the keyword. For example, if a keyword is followed by a negative word, the keyword should be assessed as absent. In the preliminary study, we evaluated the suitable character length between a keyword and a negative word. Ultimately, a 10‐character length was too short to include negative words, and a 20‐character length was too long because it sometimes included a negative word referencing another word. Therefore, we selected a 15‐character length to check whether or not a negative word was followed by a keyword. For the diagnosis of IP, keywords that describe characteristics of the lung should be assessed. In the reports there were some words whose object was not the lung. To exclude these words, we set a rule that if a different organ name was written near a keyword, then its object was regarded as not the lung. In the preliminary study, we evaluated the suitable character length between a keyword and an organ name, resulting in a ±15‐character length in a sentence being deemed suitable for this assessment.

We needed to differentiate DIP from IP caused by other diseases. If IP occurred during the administration of a target medicine but not in the pre‐ or postadministration periods, this case is plausibly DIP. However, chest CT was not frequently performed in the patients. Therefore, we used chest X‐ray findings and SP‐D and KL‐6 data to determine the presence of IP during the period in which chest CT was not performed. Because the diagnostic accuracy of these parameters is not high, we focused on the changes in their results between periods.

The occurrence rate of DIP in cases administered Gefitinib, MTX, TS‐1, or UFT estimated by the algorithm was 6.0%, 2.3%, 1.4%, and 0.7%, respectively, and after the medical records were evaluated by the medical doctors, the rates became 4.1%, 1.0%, 0.8%, and 0.5%, respectively. The accuracy of the algorithm was 63.3% overall. According to the data included in the packaging insert, the occurrence rates are 1%‐10%, 0.1%‐5%, 0.3%, and <0.1% for Gefitinib, MTX, TS‐1, and UFT, respectively. Thus, our data for Gefitinib and MTX were in the range of the data included in the packaging insert, while our data for TS‐1 and UFT were higher.

Among the 60 cases extracted from 4028 cases as DIP, 22 were found not to have DIP. Among them, 3 misdiagnoses were due to estimation error of IP in the reports. In 13 cases, other diseases more likely caused IP. This was inevitable because the grounds of our method for the detection of DIP are only the chronological relationship between the occurrence of IP and the administration of the target medicine, which is a necessary condition but not wholly sufficient. DIP was able to be denied by detailed observation in 6 cases, with IP found to be recovered during the administration period in 4 cases and other medicines more likely the cause of DIP in 2 cases.

This method was useful for efficiently extracting candidate cases in which DIP might occur. Although this method was unable to assess the absolute risk of DIP for an individual medicine accurately, the actual risk of DIP could be said to be less than the rate proposed by this method. Furthermore, the relative risks of DIP with Gefitinib, MTX, and UFT based on the risk of TS‐1 ultimately determined by the doctors were 5.1, 1.3, and 0.7, and those obtained by this algorithm were 4.3, 1.6, and 0.5, which were almost the same with the actual relative risks. This method therefore seems able to estimate the relative risk of DIP between medicines.

The accuracy of IP estimation by this NLP might be different if this method is applied to other hospitals. However, as radiologists tend to write reports using the same keywords, this method may be valid in principle.

## 5 CONCLUSION

Using the method described in this study, we successfully detected DIP for 4 anticancer drugs using the accumulated data in the EMR. Although the cases extracted by this method included some that did not have DIP, this method can estimate the relative risk of DIP between medicines.

## DISCLOSURE

The authors declare no conflicts of interest associated with this study.

## ETHICS STATEMENT

This study protocol was approved by the Ethics Review Board of Osaka University Medical Hospital (Approval No. 13531, May 8th, 2014).
