# Supplementary material for: Screening of anticancer drugs to detect drug‐induced interstitial pneumonia using the accumulated data in the electronic medical record
Source: Pharmacol Res Perspect. 2018 Jul 12;6(4):e00421. doi: 10.1002/prp2.421 (PMC6043691; doi:10.1002/prp2.421)
Supplement: Supplementary file 1 [file PRP2-6-e00421-s001.docx]

**Pharmacology Research & Perspectives**

**Screening of anti-cancer drugs to detect drug-induced interstitial pneumonia using the accumulated data in the electronic medical record**

Yoshie Shimai^1^, Toshihiro Takeda^1*^, Katsuki Okada^1^, Shirou Manabe^1^, Kei Teramoto^1, 2^, Naoki Mihara^3^, Yasushi Matsumura^1^

1. Department of Medical Informatics, Osaka University Graduate School of Medicine

2. Division of Medical Informatics, Tottori University Hospital

3. Department of Medical Informatics, National Cancer Center Hospital

* Correspondence to: T Takeda, Department of Medical Informatics, Osaka University Graduate School of Medicine, 2-2, Yamada-Oka, Suita, Osaka 565-0871, Japan. Tel.: +81-6-6879-5900, FAX: +81-6-6879-5903, E-mail: [ttakeda@hp-info.med.osaka-u.ac.jp](mailto:ttakeda@hp-info.med.osaka-u.ac.jp)

**Supplementary Material**

Supplemental Table 1. Keywords in chest CT reports and their likelihood ratios.

| Keywords | Frequency of appearance | | | Likelihood ratio | |
| --- | --- | --- | --- | --- | --- |
|  | Interstitial pneumonia | | Total |  |  |
|  | Positive | Negative |  | Positive | Negative |
| Honeycomb | 74 | 0 | 74 | 740.00 † | 0.75 † |
| Collagen | 14 | 0 | 14 | 140.00 † | 0.95 † |
| Interstitial pneumonia | 281 | 5 | 286 | 56.20 | 0.06 |
| Traction bronchiectasis | 140 | 3 | 143 | 46.67 | 0.54 |
| Reticular | 229 | 8 | 237 | 28.63 | 0.24 |
| Diffuse | 232 | 13 | 245 | 17.85 | 0.24 |
| Reactivity | 35 | 11 | 46 | 3.18 | 0.92 |
| Convergence | 17 | 6 | 23 | 2.83 | 0.96 |
| Ground-glass | 287 | 113 | 400 | 2.54 | 0.07 |
| Cyst | 44 | 23 | 67 | 1.91 | 0.92 |
| Inspiratory | 12 | 8 | 20 | 1.50 | 0.99 |
| Infection | 16 | 14 | 30 | 1.14 | 0.99 |
| Curve linear | 80 | 71 | 151 | 1.13 | 0.96 |
| Calcification | 57 | 52 | 109 | 1.10 | 0.98 |
| Consolidation | 29 | 27 | 56 | 1.07 | 0.99 |
| Lymph node | 35 | 33 | 68 | 1.06 | 0.99 |
| Infiltration | 22 | 21 | 43 | 1.05 | 1.00 |
| Emphysema | 52 | 53 | 105 | 0.98 | 1.00 |
| Swelling | 27 | 28 | 55 | 0.96 | 1.00 |
| Nodular density | 99 | 165 | 264 | 0.60 | 1.49 |
| Band | 48 | 81 | 129 | 0.59 | 1.15 |
| Tuberculosis | 11 | 19 | 30 | 0.58 | 1.03 |
| Inflammatory | 90 | 180 | 270 | 0.50 | 1.75 |
| Heterogeneity | 5 | 11 | 16 | 0.45 | 1.02 |
| Cancer | 23 | 59 | 82 | 0.39 | 1.15 |
| Thick | 23 | 59 | 82 | 0.39 | 1.15 |
| Dot like | 16 | 46 | 62 | 0.35 | 1.12 |
| Cavity | 3 | 9 | 12 | 0.33 | 1.02 |
| Lung edema | 4 | 12 | 16 | 0.33 | 1.03 |
| Mass | 15 | 51 | 66 | 0.29 | 1.14 |
| Metastasis | 7 | 41 | 48 | 0.17 | 1.13 |
| Tumor | 2 | 13 | 15 | 0.15 | 1.04 |
| Atelectasis | 4 | 49 | 53 | 0.08 | 1.18 |

†We adopted the number of IP negative patient as 0.1, because likelihood ratio cannot calculated when the number of patients is 0.

Supplemental Table 2. Keywords in chest X-ray reports and their likelihood ratios.

| Keywords | Frequency of appearance | | | Likelihood ratio | |
| --- | --- | --- | --- | --- | --- |
|  | Interstitial pneumonia | | Total |  |  |
|  | Positive | Negative |  | Positive | Negative |
| Reticular | 106 | 6 | 112 | 30.95 | 0.21 |
| Interstitial pneumonia | 86 | 6 | 92 | 25.11 | 0.36 |
| Ground-glass | 121 | 32 | 153 | 6.62 | 0.10 |
| Dot like | 13 | 13 | 26 | 1.75 | 0.96 |
| Band | 20 | 25 | 45 | 1.40 | 0.95 |
| Curve linear | 21 | 34 | 55 | 1.08 | 0.99 |
| Permeability | 7 | 14 | 21 | 0.88 | 1.01 |
| Infiltration | 5 | 11 | 16 | 0.80 | 1.01 |
| Postoperative | 11 | 25 | 36 | 0.77 | 1.03 |
| Thick | 14 | 40 | 54 | 0.61 | 1.08 |
| Nodular density | 14 | 42 | 56 | 0.58 | 1.09 |
| Atelectasis | 3 | 11 | 14 | 0.48 | 1.03 |
| Calcification | 2 | 10 | 12 | 0.35 | 1.03 |
| Metastasis | 2 | 10 | 12 | 0.35 | 1.03 |
| Inflammatory | 8 | 58 | 66 | 0.24 | 1.25 |

Supplemental Figure 1. Method for addressing missing data for not performing chest CT using supportive diagnostic methods.

**
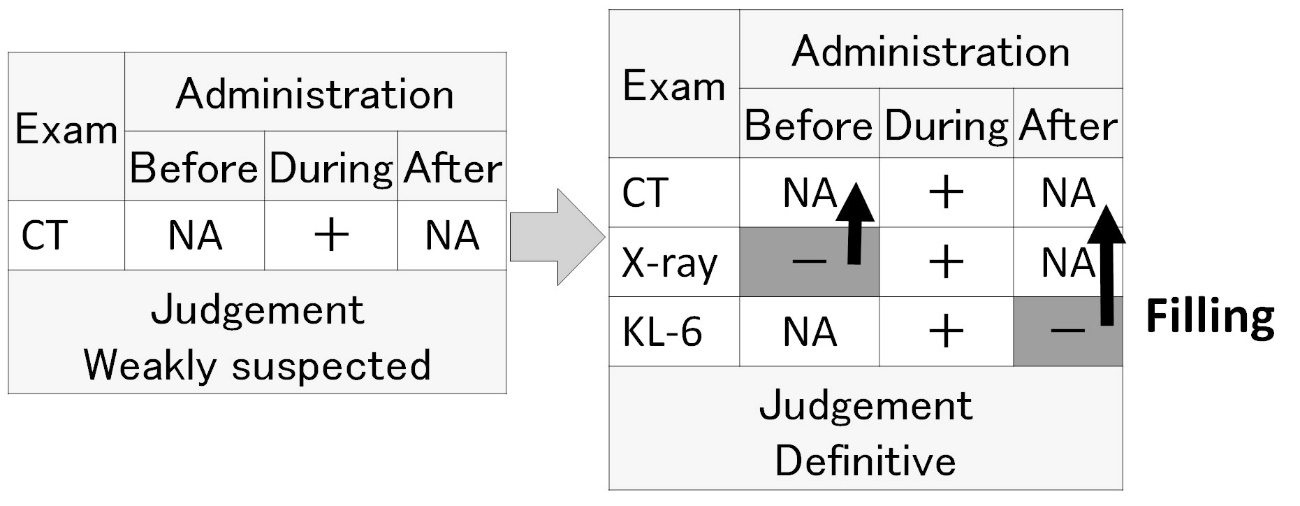
**

To solve for missing data, chest X-ray, KL-6 and SP-D data were used. For cases in which CT was only performed during the administration period, if the supportive diagnostic methods showed positive findings during the same periods and negative findings during another period, the negative diagnosis was adopted to fill in the missing data.
