# Supplementary figures and images for: Screening of anticancer drugs to detect drug‐induced interstitial pneumonia using the accumulated data in the electronic medical record
Source: Pharmacol Res Perspect. 2018 Jul 12;6(4):e00421. doi: 10.1002/prp2.421 (PMC6043691; doi:10.1002/prp2.421)

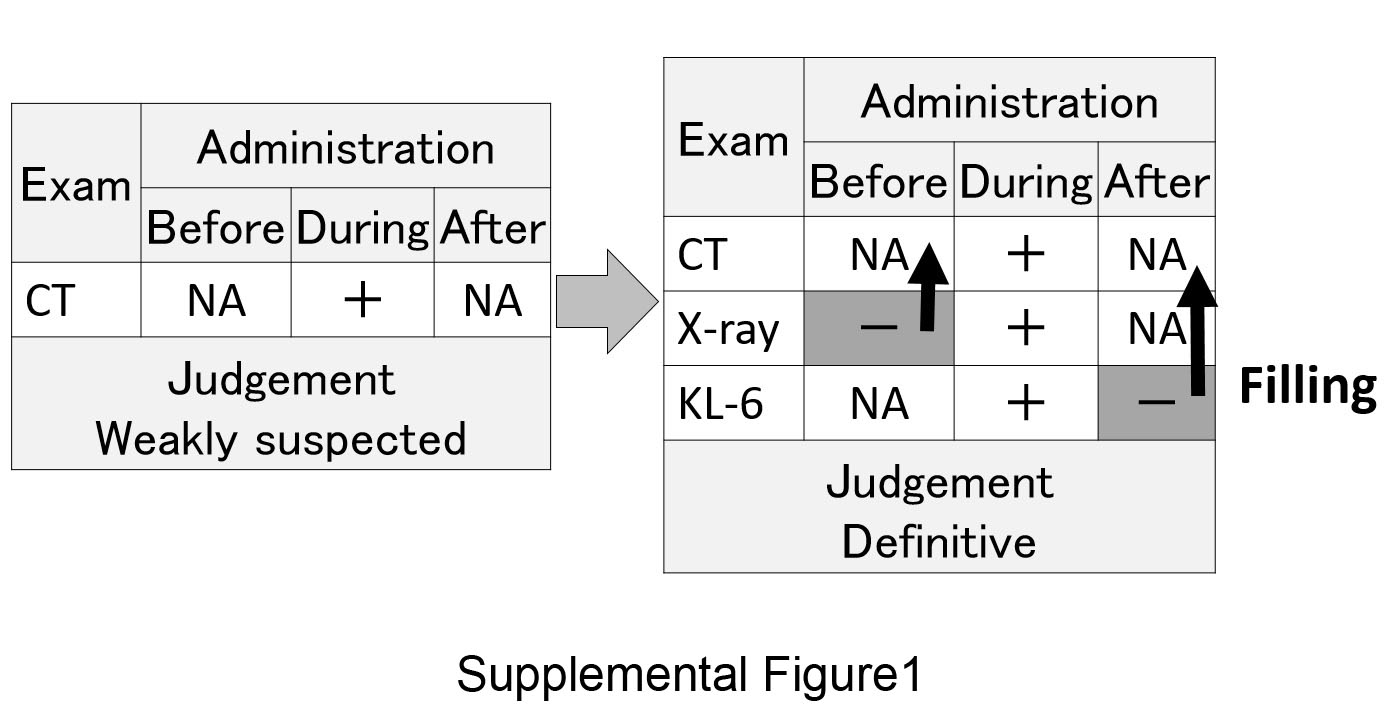

Supplement: Supplementary file 2 [file PRP2-6-e00421-s002.jpg]
